# In Vitro Suppression Effects of *Ephedra przewalskii* Stapf-Derived Natural Compounds on SARS-CoV-2

**DOI:** 10.3390/nu17182958

**Published:** 2025-09-15

**Authors:** Xiaolan Zhu, Abeer Mohamed Abdelfattah Elsayed, Masaki Kakimoto, Sachiko Sugimoto, Takemasa Sakaguchi, Keiko Ogawa-Ochiai

**Affiliations:** 1 Division of Integrated Health Sciences, Graduate School of Biomedical and Health Sciences, Hiroshima University, 1-2-3 Kasumi, Minamiku, Hiroshima 734-8551, Japan; zxl03220928@gmail.com; 2 Department of Virology, Graduate School of Biomedical and Health Sciences, Hiroshima University, 1-2-3 Kasumi, Minamiku, Hiroshima 734-8551, Japan; abeermohamed@alexu.edu.eg (A.M.A.E.); tsaka@hiroshima-u.ac.jp (T.S.); 3 Department of General Internal Medicine, Hiroshima University Hospital, 1-2-3 Kasumi, Minamiku, Hiroshima 734-8551, Japan; mkakimot@hiroshima-u.ac.jp; 4 Department of Community Based Medical System, School of Medicine, Hiroshima University, 1-2-3 Kasumi, Minamiku, Hiroshima 734-8551, Japan; 5 Faculty of Pharmacy, Juntendo University, 6-8-1 Hinode, Urayasu, Chiba 279-0013, Japan; s.sugimoto.zh@juntendo.ac.jp; 6 Kampo Clinical Center, Hiroshima University Hospital, 1-2-3 Kasumi, Minamiku, Hiroshima 734-8551, Japan

**Keywords:** *Ephedra przewalskii* Stapf, SARS-CoV-2, IC_50_, natural compounds

## Abstract

Background: *Ephedra przewalskii* Stapf stems are a traditional Mongolian medicine commonly used to treat infectious diseases. Previous in vitro experiments have shown that the extract powder derived from its stems possesses antiviral activity. However, the active compounds responsible for this activity in *E. przewalskii* Stapf have not yet been identified or evaluated. This study aimed to identify the active components in *E. przewalskii* that exhibit antiviral effects against SARS-CoV-2 in vitro and validate their antiviral activity. Methods: *E. przewalskii* stem extracts were subjected to high-performance liquid chromatography with varying methanol ratios in the mobile phase to obtain fractions with different polarities. Antiviral activity was assessed by infecting VeroE6/TMPRSS2 cells with the SARS-CoV-2 Delta strain and treating them with the obtained fractions. Infectious titers were measured using the 50% tissue culture infective dose (TCID_50_) method, and half-maximal inhibitory concentration (IC_50_) values were calculated for each fraction. The active components in the two fractions with the highest antiviral activity were identified and structurally characterized by nuclear magnetic resonance analysis. The antiviral activity of these compounds was confirmed by adding them to SARS-CoV-2-infected cells and measuring their infectious titers using the TCID_50_ method. The IC_50_ values were also calculated. Viral-particle inactivation assays were conducted by mixing the extracts with SARS-CoV-2 and measuring infectious titers. Results: (−)-Catechin, (+)-epigallocatechin-(2α→*O*→7,4α→8)-(−)-epicatechin, and *ent*-epicatechin-(4α→8;2α→*O*→7)-catechin were isolated from *E. przewalskii*. These compounds exhibited significant antiviral activity against SARS-CoV-2 but demonstrated minimal direct virucidal effects. Conclusion: (−)-Catechin, (+)-epigallocatechin-(2α→*O*→7,4α→8)-(−)-epicatechin, and *ent*-epicatechin-(4α→8;2α→*O*→7)-catechin exhibit antiviral activity against SARS-CoV-2 in infected cells.

## 1. Introduction

Owing to the ongoing mutation of SARS-CoV-2 and its continued global spread, COVID-19 remains a public health issue. As of 29 December 2024, 777,126,421 confirmed COVID-19 cases, including 7,079,925 deaths, have been reported to the World Health Organization [1]. Currently, effective antiviral drugs targeting SARS-CoV-2 are limited, and most available treatments are expensive. Consequently, the discovery of novel antiviral agents and the exploration of alternative therapeutic approaches, including drug repurposing and adjunct therapies, are urgent endeavors.

In Xinjiang, China, Chakkanda is the brand name for ephedrine-free herbal medicine derived from the plant *Ephedra przewalskii* Stapf [2,3]. According to traditional Mongolian medicine, the terrestrial stems of the plant, when boiled in water, are used to treat the common cold and influenza [4]. Previous studies have shown that crude extracts of *E. przewalskii* exhibit antiviral activity against various SARS-CoV-2 strains in vitro [5]. Furthermore, its antiviral efficacy exceeded that of *E. sinica*, a widely used herbal medicine in Kampo formulations. Phytochemical analyses of *E. przewalskii* stems revealed the presence of diverse bioactive compounds, including alkaloids, flavonoids, and flavonoid glycosides [6]. Notably, in vitro studies have demonstrated that macromolecular condensed tannins derived from *E. sinica*—formed by the polymerization of catechin units—exhibit potent inhibitory activity against SARS-CoV-2 [7]. In addition, catechin derivatives from tea have shown strong antiviral effects against SARS-CoV-2 in vitro [8]. Furthermore, molecular docking and in vitro assays have revealed that (–)-epicatechin-3-O-gallate, (–)-gallocatechin-3-O-gallate, (–)-epigallocatechin-3-O-gallate, and procyanidin B2 display varying degrees of inhibitory activity against the main protease (Mpro), which is likely attributable to the presence of galloyl groups in their molecular structures [9].

This study aimed to identify the components of *E. przewalskii* that demonstrate antiviral activity and assess their antiviral efficacy. We separated the crude extract of *E. przewalskii* into fractions of different polarities using high-performance liquid chromatography (HPLC) by adjusting the methanol ratio in the mobile phase. The antiviral effects of these fractions were evaluated, and the fractions with the highest antiviral activity were subjected to structural analysis. Finally, the isolated and identified components were evaluated for their antiviral activity in vitro and viral-particle inactivation ability.

## 2. Materials and Methods

### 2.1. Plant Materials

On 28 June 2011, a sample of *E. przewalskii* Stapf was purchased from a local pharmacy in Hotan, Xinjiang Uygur Autonomous Region, China (Voucher Number: 8042). The plant sample was previously identified as *E. przewalskii* Stapf by verifying its internal stem morphology and DNA analysis of its internal transcribed spacer region [2]. The plant’s name was further validated on 14 January 2025, using The Plant List [10]. Stem samples of the specimen were preserved in the Medicinal Botanical Garden, Faculty of Pharmaceutical Sciences, Kanazawa University.

Following a previously described method [5,11], the dried terrestrial stems of *E. przewalskii* were chopped, frozen in liquid nitrogen, and pulverized. The pulverized material was extracted with hot water (95–98 °C) at 10 times the volume-to-weight ratio for 60 min. The extract was then filtered through a No. 1 filter paper (Kiriyama Glass Works Corporation, Tokyo, Japan). The resulting extract was concentrated under reduced pressure, freeze-dried, ground into a powder, and further dried in warm air at approximately 50 °C for 60 min.

### 2.2. Virus and Cells

The VeroE6/TMPRSS2 cells used in this study (African green monkey kidney-derived cells expressing human TMPRSS2) were purchased from the Japanese Collection of Research Bioresources Cell Bank (JCRB Cell Bank, JCRB1819). The cells were cultured in Dulbecco’s modified Eagle’s medium (DMEM; FUJIFILM Wako Pure Chemical Corporation, Osaka, Japan) supplemented with 10% fetal bovine serum (Biosera, Kansas City, MO, USA), penicillin G (100 units/mL; Meiji Seika Pharma, Tokyo, Japan), and streptomycin (100 μg/mL; Meiji Seika Pharma) at 37 °C in 5% CO_2_, according to a previously described method [12].

The SARS-CoV-2 Delta variant (AY.29, Hiroshima-C77/2021 GISAID: EPI_ISL_6316561; GenBank: OP659001) used in this study was analyzed by whole-genome next-generation sequencing. A viral suspension was prepared by infecting VeroE6/TMPRSS2 cells with the virus, followed by incubation in DMEM. When cytopathic effects (CPEs) were fully observed, the supernatant was collected, centrifuged (1000× *g* for 5 min at 20 °C), and filtered through a 0.45 μm filter (Kurabo Industries Ltd., Osaka, Japan). The viral titer was determined using the standard 50% tissue culture infective dose (TCID_50_) method. Briefly, the virus was serially diluted 10-fold, inoculated into cells in 96-well plates, and incubated for 7 d to assess CPEs. Each dilution was tested in quadruplicate or octuplicate, and viral titers were measured for each experimental run. Infectious titers were calculated from the results and expressed as TCID_50_/mL, as previously described [5].

### 2.3. Extraction Procedures

#### 2.3.1. Separation of Complex Compounds from *E. przewalskii*

Following the method reported by Wang et al. [13], column chromatography was conducted using a Cosmosil 75C18-OPN column (Nacalai Tesque, Kyoto, Japan). High-performance liquid chromatography (HPLC) was carried out on an Inertsil ODS column (GL Sciences, Tokyo, Japan; Φ 10 mm × 250 mm).

The solid extract (powder) of *E. przewalskii* (2.3 g; prepared as described in Section 2.1) was separated by reversed-phase open column chromatography (ODS, Φ = 30 mm, L = 13 cm) with gradient elution as follows: MeOH:H_2_O = 0:1, 0.2 L; MeOH:H_2_O = 1:9, 0.2 L; MeOH:H_2_O = 1:5, 0.2 L; MeOH:H_2_O = 3:7, 0.2 L; MeOH:H_2_O = 2:3, 0.2 L; MeOH:H_2_O = 1:1, 0.2 L; MeOH:H_2_O = 1:0, 0.2 L. This process yielded seven subfractions, which were labeled Fractions 1–7. Fraction 3 (312.0 mg) was purified by HPLC (MeOH:H_2_O = 1:4, 2 mL/min) to give (–)-catechin (yield: 0.6 mg; hereafter referred to as Component-1) from the peak with a retention time of 20 min. Fraction 5 (402.3 mg) was purified by HPLC (MeOH:H_2_O (2:3)–0.01% TFA, 2 mL/min) to give (+)-epigallocatechin-(2α→*O*→7,4α→8)-(−)-epicatechin (yield: 4.3 mg; hereafter referred to as Component-2) and *ent*-epicatechin-(4a→8;2a→*O*→7)-catechin (yield: 3.1 mg; hereafter referred to as Component-3) from the peaks with retention times of 12 and 14 min, respectively. The compounds in these fractions were identified using previously published data [14,15,16].

#### 2.3.2. Dissolution of Powders Obtained from Fractions 1–7

The powders obtained from Fractions 1–7 were dissolved separately in DMEM(−) and DMEM(−) containing 1% DMSO to investigate the potential presence of lipophilic substances in different fractions. The solutions were incubated at 50 °C for 60 min, after which their concentrations were adjusted.

### 2.4. Extraction, Separation, and Identification of E. przewalskii Antiviral Components

#### 2.4.1. Identification of Antiviral-Active Fractions

VeroE6/TMPRSS2 cells were seeded in 96-well plates at 100% confluency and 50 µL of the virus solution was added at a multiplicity of infection (MOI) of 0.05. After 2 h of adsorption, the virus solution was removed, and the cells were further incubated with the different fractions. DMEM(−) served as the blank control (0 mg/mL) for the antiviral assay of samples dissolved in DMEM(−).The maximum concentration tested was based on the results of the cytotoxicity assay and the concentration at which all materials dissolved. The minimum concentration tested was based on the results of the preliminary experiment and the concentration corresponding to the same infectious titer as the negative control. DMEM(−) served as the negative control for the water-soluble groups, whereas DMEM with 1% DMSO served as the negative control for the lipophilic groups. Fractions were tested at concentrations selected based on their solubility in each medium and restricted to conditions with ≤5% cytotoxicity.

In DMEM(−) (mg/mL): Fraction 1: 1.25–2.5, Fraction 2: 0.08–2.5, Fraction 3: 0.02–0.08, Fraction 4: 0.005–0.02, Fraction 5: 0.04–0.16, Fraction 7: 0.04–0.16, Fraction 7: 0.0025–0.005. In DMEM with 1% DMSO (mg/mL): Fraction 1: 0.04–2.5, Fraction 2: 0.04–2.5, Fraction 3: 0.02–0.08, Fraction 4: 0.005–0.02, Fraction 5: 0.01–0.04, Fraction 6: 0.01–0.08, Fraction 7: 0.0025–0.01. The concentrations of each fraction are listed in Supplementary Table S1.

After 24 h, the culture supernatants were collected and tested for viral infectivity using the TCID_50_ method, as previously described [5]. Viral titers and fraction concentrations were plotted on a logarithmic scale, and an approximate linear regression curve was fitted to calculate the half-maximal inhibitory concentration (IC_50_), as described by Nomura et al. [17].

#### 2.4.2. Identification of Antiviral-Active Compounds in the Active Fractions

The *E. przewalskii* fractions with significant antiviral effects were selected for further analysis. Following a previously reported method [13], Component-1, -2, and -3 were subjected to positive- and negative-ion high-resolution electrospray ionization mass spectrometry using an LTQ Orbitrap XL spectrometer (Thermo Fisher Scientific, Waltham, MA, USA). The identity of the compounds was confirmed based on their ^1^H and ^13^C nuclear magnetic resonance spectra, which were recorded on an Avance III HD spectrometer (Bruker, Billerica, MA, USA) at 700 and 150 MHz, respectively. The residual solvent signal served as a reference. The absolute configurations of the compounds were determined by observing their specific rotations using a P-1030 spectropolarimeter (JASCO, Tokyo, Japan).

### 2.5. Cytotoxicity Assay

VeroE6/TMPRSS2 cells were incubated for 24 h in DMEM containing specific concentrations of the fractions. Fractions were assayed at stepwise concentrations selected by solubility and limited to ≤5% cytotoxicity: in DMEM(−) (mg/mL): Fraction 1–7: 0.01–2.5; In DMEM with 1% DMSO (mg/mL): Fraction 1–2: 0.01–2.5, Fraction 3: 0.01–0.08, Fraction 4: 0.005–0.02, Fraction 5: 0.005–0.08, Fraction 6: 0.01–0.16, Fraction 7: 0.01 and 0.08. Serial dilutions of the *E. przewalskii* fractions were prepared as listed in Supplementary Table S2.

Cytotoxicity was assessed using a lactate dehydrogenase (LDH) cytotoxicity assay kit (LDH-WST; Dojindo Laboratories, Kumamoto, Japan). The LDH released from the cells into the medium was measured colorimetrically at 490 nm using a TriStar LB 941 plate reader (Berthold Technologies, Wildbad, Germany).

The cytotoxicity of the fractions was calculated based on the absorbance, as described by Kakimoto et al. [5]. The cytotoxicity of the high control group (cells lysed with surfactant) was set to 100%, whereas that of the low control group [DMEM(−) with 1% DMSO and DMEM(−)] was set to 0%. To ensure consistency and interpretability of the results, we adjusted any value indicating cytotoxicity exceeding 100% to 100%. The experimental setup was established according to the manufacturer’s instructions (Dojindo Molecular Technologies).

### 2.6. Replication of SARS-CoV-2

VeroE6/TMPRSS2 cells were seeded in 96-well plates at 100% confluency, and 50 µL of the virus solution was added at an MOI of 0.05. After 2 h of adsorption, the inoculated virus solution was removed, and the cells were further incubated with different fractions.

The antiviral effect of specific fractions was assayed at the following concentrations: Component-1: 0.02, 0.04, 0.06, 0.08, 0.1, and 0.12 mg/mL; Component-2: 0.02, 0.04, 0.08, 0.12, 0.16, and 0.2 mg/mL; Component-3: 0.02, 0.04, 0.06, 0.08, 0.1, and 0.12 mg/mL. In this experiment, DMEM(−) served as the blank control (0 mg/mL). The selection criteria for the test concentrations were based on the conditions described in Section 2.4.1; however, the fractions were dissolved in DMEM(−) only. The TCID_50_ and IC_50_ values were calculated as described in Section 2.4.1.

### 2.7. Viral-Particle Inactivation Assay

A solution of each compound (90 µL) was mixed with the viral solution and incubated at 20–25 °C for 3 min. After 7 d of incubation, the infectivity of the virus solution was assessed using the TCID_50_ method. The concentration of the solution was selected such that its cytotoxicity remained below 5%. Phosphate-buffered saline (PBS) was used as the blank control, whereas a 70% EtOH solution served as the viral-particle inactivation control.

### 2.8. Statistical Analysis

The effects of the extracted compounds on SARS-CoV-2 replication and viral particle inactivation were evaluated using the Behrens–Kärber method [18]. The relationship between compound concentration and viral titer was analyzed using log-linear regression and Microsoft Excel (version 16.77.1). IC_50_ values were calculated according to the method described by Kakimoto et al. [5].

To compare the direct inactivation effects of Component-1, -2, and -3 on the virus, we analyzed the data via unpaired *t*-tests using GraphPad Prism (version 10.4.1; GraphPad Software, Boston, MA, USA, www.graphpad.com). The control and experimental groups had sample sizes of 4. A significance threshold (cutoff *p*-value) of ≤0.05 (*) was adopted for all statistical analyses.

## 3. Results

### 3.1. Separation of E. przewalskii

Substances with different polarities were separated into different fractions by varying the MeOH ratio of the mobile phase. The HPLC chromatogram of the extract is shown in Supplementary Figure S1.

### 3.2. Antiviral Effects of the E. przewalskii Fractions and Their Cytotoxicity to Vero/TMPRSS2 Cells

The cytotoxicity of Fractions 1–7 and Component-1, -2, and -3 was assessed using the LDH assay. Among the *E. przewalskii* fractions dissolved in DMEM(−), Fractions 1 and 2 exhibited a cytotoxicity of <5%, even at the highest concentration tested (2.5 mg/mL). Fractions 3, 5, and 6 exhibited ≤ 5% cytotoxicity at concentrations of up to 0.16 mg/mL. Fraction 4 showed ≤ 5% cytotoxicity at concentrations below 0.02 mg/mL, while Fraction 7 displayed ≤ 5% cytotoxicity at concentrations below 0.04 mg/mL.

Among the *E. przewalskii* fractions dissolved in DMEM with 1% DMSO, Fractions 1 and 2 also exhibited < 5% cytotoxicity at the highest concentration tested (2.5 mg/mL). Fractions 3 and 6 demonstrated ≤ 5% cytotoxicity at concentrations of up to 0.08 mg/mL. Fractions 4, 5, and 7 exhibited ≤ 5% cytotoxicity at concentrations of ≤0.02, ≤0.08, and ≤0.01 mg/mL, respectively.

**Figure 1:**
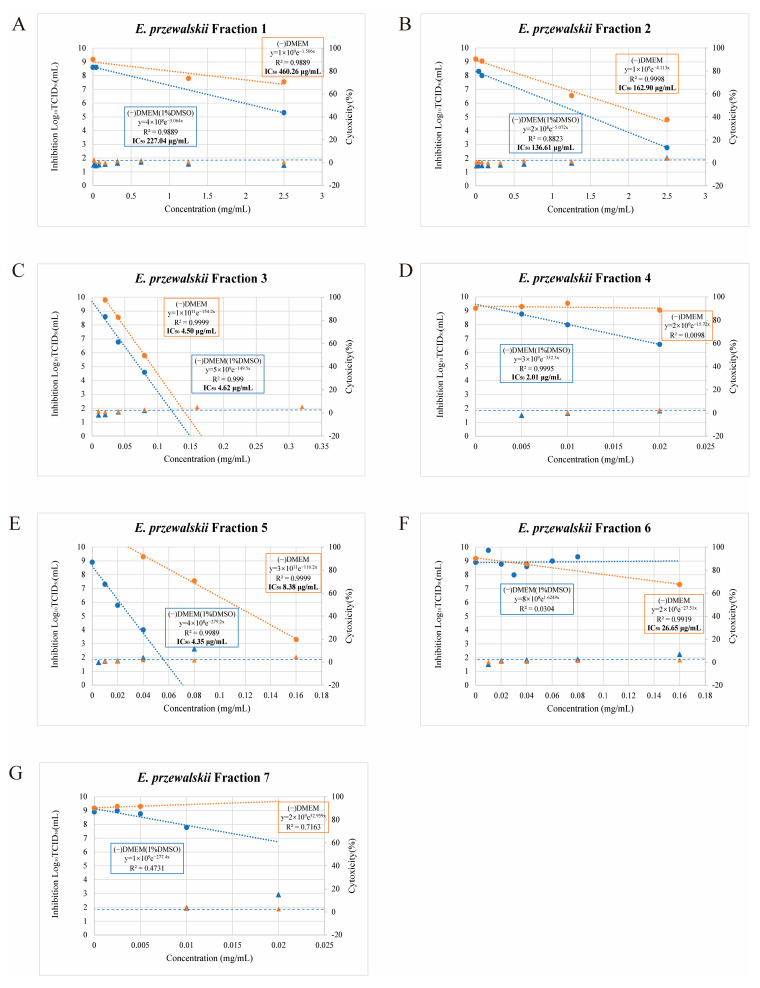
Evaluation of the cytotoxicity of *E. przewalskii* Fractions 1–7 (**A**–**G**) and their antiviral effects against SARS-CoV-2 under multiplicities of infection (MOIs) of 0.05 and 10. (**A**) Fraction 1, (**B**) Fraction 2, (**C**) Fraction 3, (**D**) Fraction 4, (**E**) Fraction 5, (**F**) Fraction 6, (**G**) Fraction 7. The antiviral effects of the fractions were compared in DMEM(−) and DMEM (1% DMSO). The orange points and lines represent DMEM(−) while the blue ones represent DMEM (1% DMSO). Triangles and circles indicate the cytotoxicity and infectious titer at a given concentration, respectively. Approximate equations, determination coefficients (*R*^2^), and half-maximal inhibitory concentrations (IC_50_) for each MOI are provided.

The IC_50_ values for the *E. przewalskii* fractions dissolved in DMEM(−) and DMEM with 1% DMSO are shown in Figure 1. IC_50_ values could not be calculated for Fractions 4 and 7 dissolved in DMEM(−) (Figure 1D,G) and Fractions 6 and 7 dissolved in DMEM with 1% DMSO (Figure 1F,G) owing to their lack of antiviral activity. The IC_50_ values are listed in Table 1.

### 3.3. Structures of Isolated Compounds

**Figure 2:**
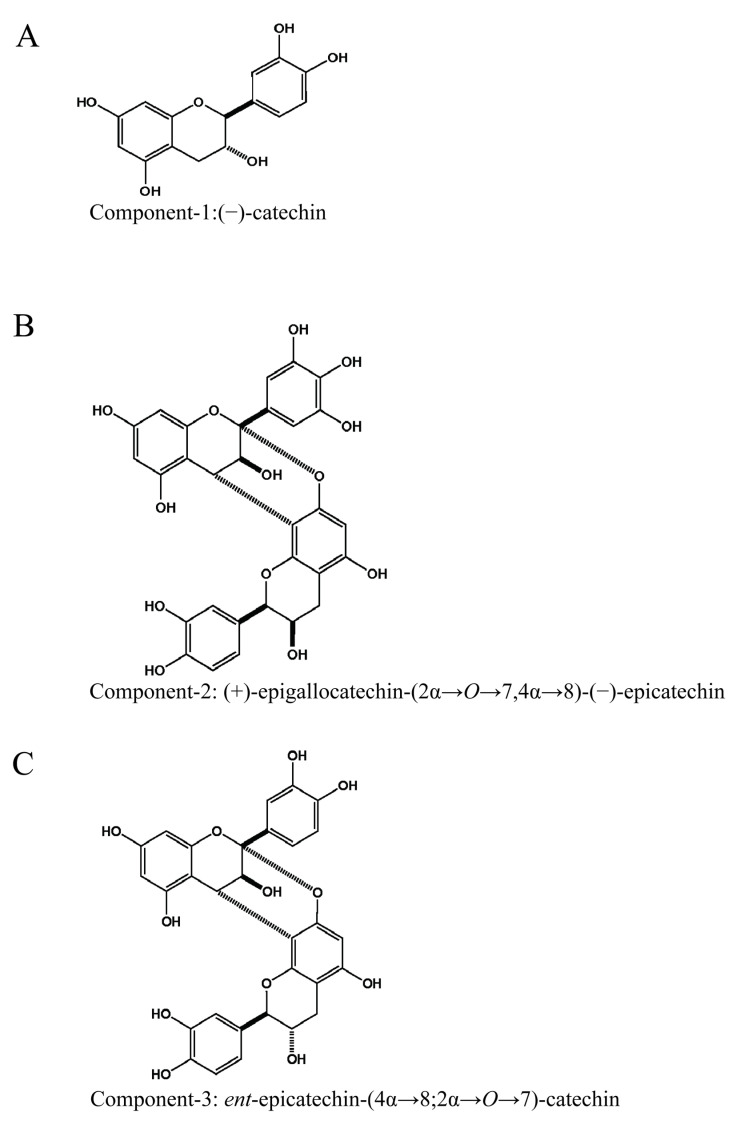
Structures of the components isolated from *E. przewalskii* Fractions 3 and 5. (**A**) Component-1: (−)-catechin, (**B**) Component-2: (+)-epigallocatechin-(2α→*O*→7,4α→8)-(−)-epicatechin, and (**C**) Component-3: *ent*-epicatechin-(4α→8;2α→*O*→7)-catechin.

The structures of Component-1, -2, and -3 are presented in Figure 2.

### 3.4. Antiviral Effects of Component-1, -2, and -3

**Figure 3:**
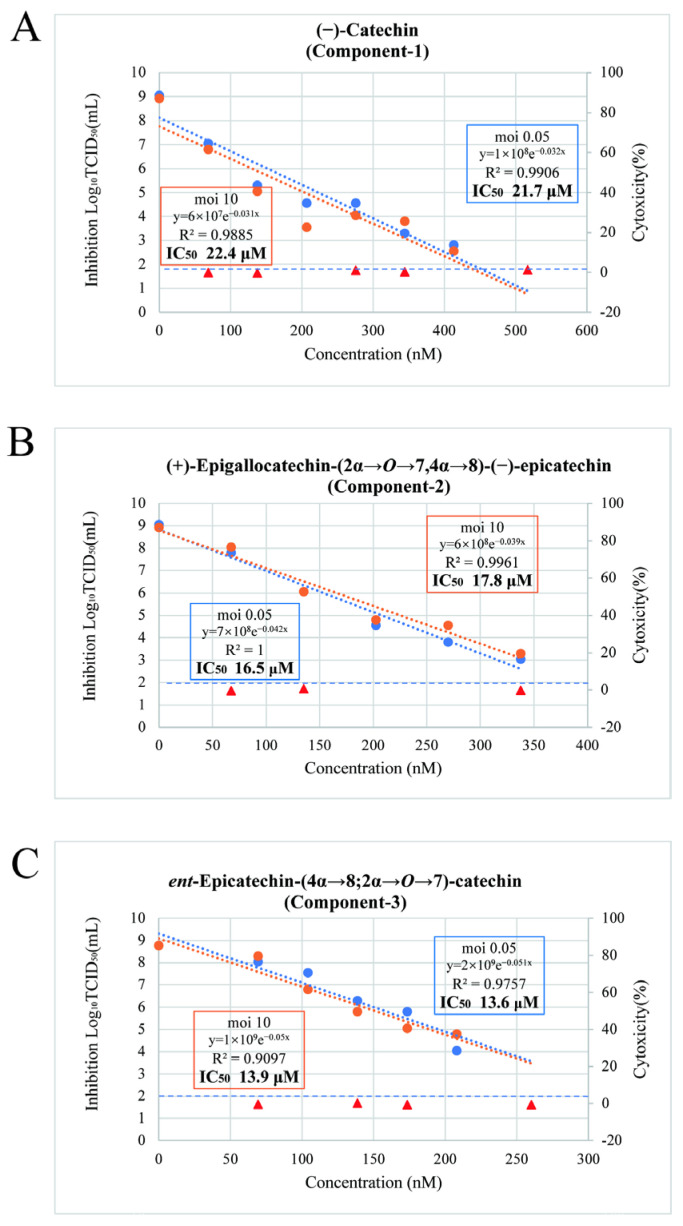
Evaluation of the cytotoxicity of Component-1, Component-2, and Component-3 and their antiviral effects against SARS-CoV-2 under multiplicities of infection (MOIs) of 0.05 and 10. (**A**) Component-1, (**B**) Component-2, (**C**) Component-3. Orange plots represent the condition of MOI 10, and blue plots represent the condition of MOI 0.05. Triangles indicate the cytotoxicity at a given concentration. Circles indicate the infectious titer at a given concentration. Approximate equations, determination coefficients (*R*^2^), and half-maximal inhibitory concentrations (IC_50_) are provided for each MOI. The antiviral effects of all fractions were nearly identical under both conditions.

Cells were infected with SARS-CoV-2 at MOIs of 0.05 and 10 and then treated with the fractions. The IC_50_ of Component-1 was 21.6 μM at an MOI of 0.05 condition and 22.4 μM at an MOI of 10. The IC_50_ of Component-2 was 16.5 μM at an MOI of 0.05 condition and 17.8 μM at an MOI of 10. The IC_50_ of Component-3 was 13.6 μM at the MOI 0.05 condition and 13.9 μM at the MOI 10. These results indicate that the IC_50_ values of these compounds were highly similar under both MOIs (Figure 3).

### 3.5. Viral-Particle Inactivation Effects of Component-1, Component-2, and Component-3

**Figure 4:**
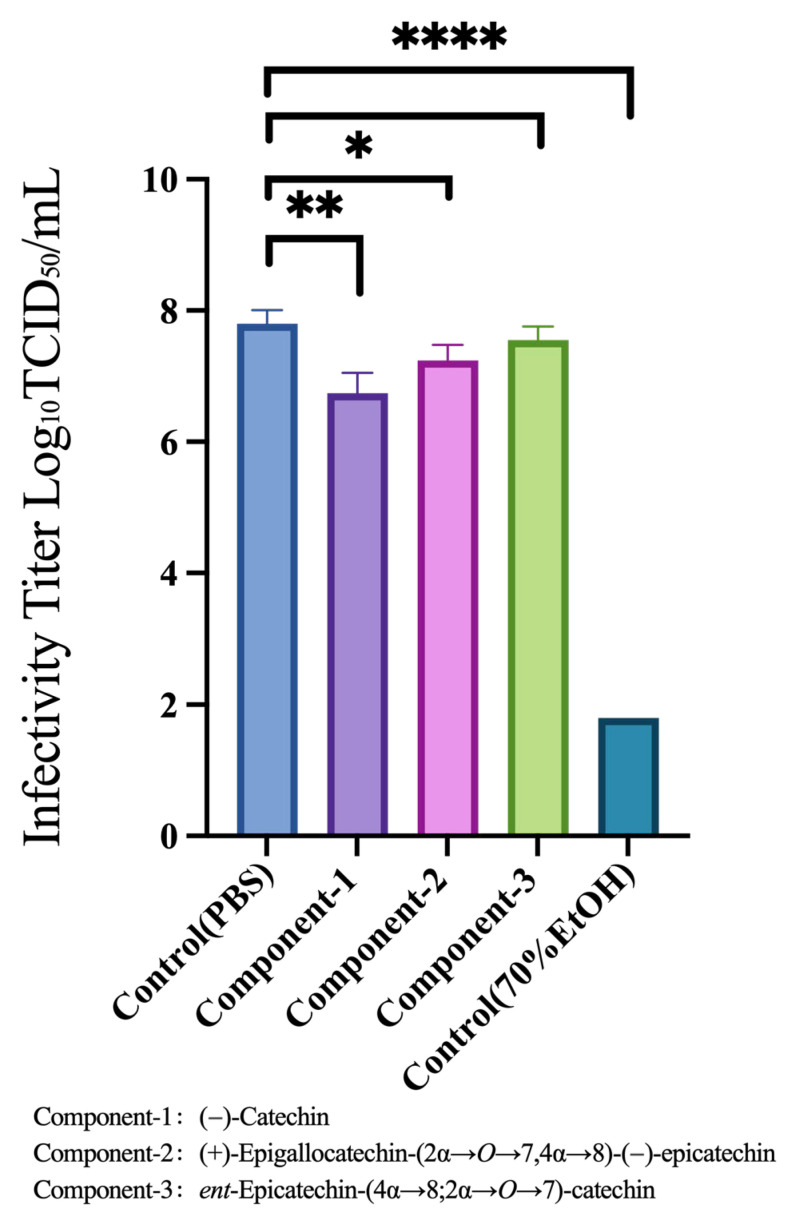
Viral-particle inactivating effect of Component-1, Component-2, and Component-3. Each group had a sample size of 4. The results for the four groups were analyzed against those of the PBS group using an unpaired *t* test.

Based on the criteria of cytotoxicity within 5%, absence of insoluble substances, and antiviral replication effects described in Section 3.2, we further tested Component-1, Component-2, and Component-3 at concentrations of 0.12, 0.2, and 0.12 mg/mL, respectively. These compounds were mixed with the viral stock solution to analyze their infection titers and assess their ability to inactivate viral particles. Compared with the PBS control group, the compounds demonstrated minimal reductions in the infection titer, indicating a weak viral inactivation effect (Figure 4).

The results for the four groups were analyzed against those of the PBS group using an unpaired *t* test. Significant differences were observed between the PBS and EtOH groups (*p* < 0.0001), PBS and Component-1 groups (*p* = 0.0013), and PBS and Component-2 groups (*p* = 0.0117). No significant difference was observed between the PBS and Component-3 groups (*p* = 0.1340).

## 4. Discussion

The integration of traditional medicine with modern science has immense potential for diverse applications. This approach, which is built on generations of experiential knowledge, can advance the field and lead to broader and more scientifically grounded applications.

In our study, we compared the fractions separated from the extract of *E. przewalskii* stems via HPLC and found that Fractions 3 and 5 exhibited stronger antiviral effects than the other fractions and the crude *E. przewalskii* extract. These fractions were found to contain compounds with significant antiviral activity. Subsequently, we isolated and identified the following compounds from Fractions 3 and 5: Component-1: (–)-catechin, Component-2: (+)-epigallocatechin-(2α→*O*→7,4α→8)-(−)-epicatechin, and Component-3: *ent*-epicatechin-(4α→8;2α→*O*→7)-catechin. These compounds have demonstrated pronounced inhibitory effects against SARS-CoV-2. Furthermore, we found that under MOI 0.05 conditions, the IC_50_ values of the three compounds were all lower than that of the *E. przewalskii* extract, indicating that the antiviral effects of the three compounds were superior to those of the *E. przewalskii* extract [5].

Catechin is a polyphenolic compound widely found in many plants. Previous studies have primarily focused on (+)-catechin structure, demonstrating its antioxidant and anti-inflammatory properties [19]. Researchers have also extracted and isolated (+)-catechin from green tea and validated its antiviral effects, particularly against TGEV [20] and influenza viruses [21].

In this study, we confirmed the inhibitory effects of the Component-1 structure isolated from *E. przewalskii* on SARS-CoV-2. Component-2 and Component-3 belong to a class of compounds called tannins. Tannins are abundant in the stems of many *Ephedra* species and have been extensively studied for their diverse bioactivities, including antibacterial, antiviral, anti-inflammatory, and anticancer properties [15]. Previous studies have shown that tannins from *Ephedra* exhibit significant inhibitory effects against influenza viruses [22,23]. Moreover, molecular docking studies have suggested that tannins may inhibit the main protease activity of SARS-CoV-2 [24]. These findings underscore the potential antiviral activity of individual compounds within the extracts, providing a theoretical basis for drug development. Previous studies have reported that *E. przewalskii* extracts exhibit significant direct viral inactivation [5]. However, in this study, Component-2 showed limited viral-particle inactivation effects, whereas Component-3 did not. Combining these findings with the observation that all three compounds exhibited nearly identical antiviral effects under two different MOI conditions, 0.05 (low MOI) and 10 (high MOI), where at high MOI most cells are infected simultaneously, the observed antiviral effects suggest that the compounds exert their activity post-entry and inhibit intracellular viral replication. This indicates that these compounds can penetrate host cells and function in the intracellular environment. Conversely, under low MOI (0.05), where only a small fraction of cells are initially infected and viral spread occurs via progeny virions, the inhibition of secondary infection suggests that, in addition to suppressing intracellular replication, the compounds may interfere with progeny virus release and subsequent reinfection of neighboring cells. In future studies, we will prioritize mechanistic investigations targeting inhibition of the main protease, the RNA-dependent RNA polymerase (RdRp), and additional factors operative during the viral replication cycle, including viral mRNA synthesis.

Although our study provides new insights into the pharmacological properties of these three active compounds for viral inhibition, we must acknowledge certain methodological limitations. According to traditional medicinal records, the stems of *E. przewalskii* Stapf are boiled in water, and the decoction is orally administered [4]. Although Tsumura et al. did not disclose their extraction methods, the *E. przewalskii* extract obtained using their method was highly comparable with previous reports [5,11]. Thus, we believe that methodological differences among different studies exerted minimal influence on the pharmacological efficacies reported in this work.

Although there is no clear definition for considering cytotoxicity, in this study we followed previous reports [5] and set the threshold for cytotoxicity at below 5% when determining the antiviral effects.

In this study, IC_50_ values were determined using the TCID_50_ method, and the calculated values represent statistically processed estimates. While increasing the number of replicates and reporting standard deviations would enhance statistical reliability, the limited availability of *E. przewalskii* samples made it impractical to provide standard deviations in this study.

Owing to the very small quantities of Component-1, Component-2, and Component-3 obtained during isolation and purification, significant losses occurred during the filtration process in this study. Moreover, when preparing the test solutions, we relied on visual observation to dilute the *E. przewalskii* extract powders to a concentration without visible precipitation. However, we believe that these factors had minimal effect on the results.

As our findings are based on in vitro cellular experiments, further studies should be conducted to explore the mechanisms by which Component-1, Component-2, and Component-3 inhibit viruses in cells. Animal experiments and clinical studies are also necessary to evaluate the oral bioavailability, hepatotoxicity, nephrotoxicity, and clinical efficacy of these compounds, thus facilitating more comprehensive testing.

## 5. Conclusions

The compounds derived from *E. przewalskii* that exhibited antiviral activity against SARS-CoV-2 within infected cells were identified as (–)-catechin, (+)-epigallocatechin-(2α→*O*→7,4α→8)-(–)-epicatechin, and *ent*-epicatechin-(4α→8;2α→*O*→7)-catechin. These results suggest that catechin-derived active compounds may serve as a foundation for the development of antiviral therapies based on single compounds.

## Figures and Tables

**Table 1:** IC_50_ values (µg/mL) of *E. przewalskii* fractions 1–7 in DMEM(−) and DMEM with 1% DMSO.

Fraction	DMEM(−) (µg/mL)	DMEM with 1%DMSO (µg/mL)
1	460.3	227
2	162.9	136.6
3	4.5	4.6
4	not determined	2
5	8.4	4.4
6	26.6	not determined
7	not determined	not determined

“Not determined” indicates that the IC_50_ could not be measured under the corresponding condition. Fractions with concentrations exhibiting less than 5% cytotoxicity were selected for subsequent experiments.

## Data Availability

The data that support the findings reported herein are available from the corresponding author upon reasonable request.

## Supplementary Materials

The following supporting information can be downloaded at https://www.mdpi.com/article/10.3390/nu17182958/s1, Supplementary Figure S1. High-performance liquid chromatogram of the E. przewalskii extract. Supplementary Table S1: Concentrations used for the antiviral activity assessment of the fractions. Supplementary Table S2: Concentrations used for the cytotoxicity evaluation of the fractions.

## Abbreviations

The following abbreviations are used in this manuscript:

CPE	Cytopathic effect
DMEM	Dulbecco’s modified Eagle’s medium
DMSO	Dimethylsulfoxide
HPLC	High-performance liquid chromatography
IC_50_	Half-maximal inhibitory concentration
JCRB	Japanese Collection of Research Bioresources
LDH	Lactate dehydrogenase
MOI	Multiplicity of infection
PBS	Phosphate-buffered saline
SARS-CoV-2	severe acute respiratory syndrome coronavirus 2
TCID_50_	50% tissue culture infective dose
TGEV	Transmissible gastroenteritis virus
