# Supplementary material for: In Vitro Suppression Effects of Ephedra przewalskii Stapf-Derived Natural Compounds on SARS-CoV-2
Source: Nutrients. 2025 Sep 15;17(18):2958. doi: 10.3390/nu17182958 (PMC12473021; doi:10.3390/nu17182958)
Supplement: Supplementary file 1 [file nutrients-17-02958-s001.zip › nutrients-3854887-supplementary.pdf]

Supplementary Material

In Vitro Suppression Effects of *Ephedra przewalskii* Stapf-Derived Natural Compounds on SARS-CoV-2

Xiaolan Zhu [1], Abeer Mohamed Abdelfattah Elsayed [2], Masaki Kakimoto [3,4], Sachiko Sugimoto [5], Takemasa Sakaguchi [2] and Keiko Ogawa-Ochiai [6,*]

[1] Division of Integrated Health Sciences, Graduate School of Biomedical and Health Sciences, Hiroshima University, 1-2-3 Kasumi, Minamiku, Hiroshima 734-8551, Japan; zxl03220928@gmail.com
[2] Department of Virology, Graduate School of Biomedical and Health Sciences, Hiroshima University, 1-2-3 Kasumi, Minamiku, Hiroshima 734-8551, Japan; abeermohamed@alexu.edu.eg (A.M.A.E.); tsaka@hiroshima-u.ac.jp (T.S.)
[3] Department of General Internal Medicine, Hiroshima University Hospital, 1-2-3 Kasumi, Minamiku, Hiroshima 734-8551, Japan; mkakimot@hiroshima-u.ac.jp
[4] Department of Community Based Medical System, School of Medicine, Hiroshima University, 1-2-3 Kasumi, Minamiku, Hiroshima 734-8551, Japan
[5] Faculty of Pharmacy, Juntendo University, 6-8-1 Hinode, Urayasu, Chiba 279-0013, Japan; s.sugimoto.zh@juntendo.ac.jp
[6] Kampo Clinical Center, Hiroshima University Hospital, 1-2-3 Kasumi, Minamiku, Hiroshima 734-8551, Japan
* Correspondence: okeiko22@hiroshima-u.ac.jp; Tel.: +082-257-1921

Supplementary Table S1: Concentrations used for the antiviral activity assessment of the fractions.

Fraction	DMEM(−) (mg/mL)	DMEM(−) with 1 % DMSO (mg/mL)
1	1.25; 2.5	0.04; 2.5
2	0.08; 1.25; 2.5	0.04; 0.08; 2.5
3	0.02; 0.04; 0.08	0.02; 0.04; 0.08
4	0.005; 0.01; 0.02	0.005; 0.01; 0.02
5	0.04; 0.08; 0.16	0.01; 0.02; 0.04
6	0.04; 0.16	0.01; 0.02; 0.04; 0.06; 0.08
7	0.0025; 0.005	0.0025; 0.005; 0.01

Supplementary Table S2: Concentrations used for the cytotoxicity evaluation of the fractions.

Fraction	DMEM(−) (mg/mL)	DMEM(−) with 1 % DMSO (mg/mL)
1	0.01; 0.02; 0.08; 0.16; 0.32; 0.63; 1.25; 2.5	0.01; 0.02; 0.04; 0.08; 0.16; 0.32; 0.63; 1.25; 2.5
2	0.01; 0.02; 0.04; 0.08; 0.16; 0.32; 0.63; 1.25; 2.5	0.01; 0.02; 0.04; 0.08; 0.16; 0.32; 0.63; 1.25; 2.5
3	0.01; 0.02; 0.04; 0.08; 0.16; 0.32; 0.63; 1.25; 2.5	0.01; 0.02; 0.04; 0.08
4	0.01; 0.02; 0.04; 0.08; 0.16; 0.32; 0.63; 1.25; 2.5	0.005; 0.01; 0.02
5	0.01; 0.02; 0.04; 0.08; 0.16; 0.32; 0.63; 1.25; 2.5	0.005; 0.01; 0.02; 0.04; 0.08
6	0.01; 0.02; 0.04; 0.08; 0.16; 0.32; 0.63; 1.25; 2.5	0.01; 0.02; 0.04; 0.08; 0.16
7	0.01; 0.02; 0.04; 0.08; 0.16; 0.32; 0.63; 1.25; 2.5	0.010.08

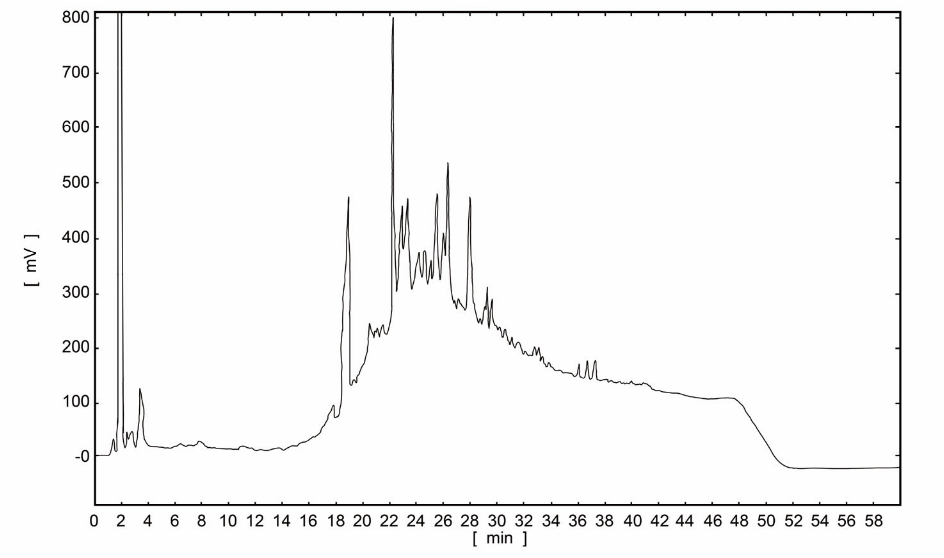

Supplementary Figure S1. High-performance liquid chromatogram of the *E. przewalskii* extract.

The presence of multiple peaks suggests the presence of various compounds in the extract.
